# Normal weight obesity and physical fitness in Chinese university students: an overlooked association

**DOI:** 10.1186/s12889-018-6238-3

**Published:** 2018-12-04

**Authors:** Meizhen Zhang, Moritz Schumann, Tao Huang, Timo Törmäkangas, Sulin Cheng

**Affiliations:** 1 Department of Physical Education, Exercise, Health and Technology Centre, Shanghai Jiao Tong University, Shanghai, 200240 China; 2 College of Physical Education, Taiyuan University of Technology, Taiyuan, Shanxi China; 3 School of Life Sciences and Biotechnology, Shanghai Jiao Tong University, Shanghai, 200240 China; 4 Department of Molecular and Cellular Sports Medicine, German Sport University, Cologne, Germany; 5 Gerontology Research Center, Faculty of Sport and Health Sciences, University of Jyväskylä, Jyväskylä, Finland; 6 The Key Laboratory of Systems Biomedicine, Ministry of Education, and Exercise Translational Medicine Center, Shanghai Center for Systems Biomedicine, Shanghai Jiao Tong University, Shanghai, China; 7 Faculty of Sport and Health Sciences, University of Jyväskylä, Jyväskylä, Finland

**Keywords:** Body-mass index, Body composition, Skeletal muscle mass, Physical activity, Public health

## Abstract

**Background:**

The primary aim of this study was to examine the associations of normal weight obesity (NWO) with physical fitness in Chinese university students. As a secondary aim, we assessed whether possible differences in physical fitness between students classified as NWO and normal weight non-obese (NWNO) were mediated by skeletal muscles mass.

**Methods:**

A total of 383 students (205 males and 178 females, aged 18–24 years) from two universities volunteered to participate in this study. Body height and weight were measured by standard procedures and body composition was assessed by bio-impedance analysis (InBody 720). NWO was defined by a BMI of 18.5–23.9 kg/m^2^ and a body fat percentage of > 20% or > 30% in male and female students, respectively. Physical fitness was measured using a 10-min intermittent endurance running test (Andersen test), countermovement jumps (CMJ) and a 5 × 5 m shuttle run test (5mSR). The level of leisure time physical activity (PA) was assessed by a questionnaire.

**Results:**

13.7% of male and 27.5% of female students were classified as NWO. Compared to NWNO, students classified as NWO showed a significantly poorer performance in the Andersen test (males: 1146 ± 70 m vs. 1046 ± 95 m, females: 968 ± 61 m vs. 907 ± 67 m, *p* < 0.001), CMJ (males: 55.0 ± 7.6 cm vs. 44.9 ± 7.5 cm, females: 39.8 ± 8.0 cm vs. 33.7 ± 5.9 cm, *p* < 0.001) and 5mSR (males: 18.7 ± 1.0 s vs. 20.0 ± 0.9 s, females: 21.1 ± 1.1 s vs. 22.4 ± 1.3 s, *p* < 0.001), respectively. The lower levels of physical fitness in NWO were partially explained by lower skeletal muscle mass (*p* < 0.001) both in male and female students.

**Conclusions:**

NWO was associated with poorer physical fitness and the relationship was partially mediated by lower skeletal muscle mass. The study indicated that attention should be paid for the potential hidden health risk in university students with normal body mass index but excessive fat mass.

**Electronic supplementary material:**

The online version of this article (10.1186/s12889-018-6238-3) contains supplementary material, which is available to authorized users.

## Background

The prevalence of obesity is nowadays one of the most severe health concerns worldwide, due to the often observed complications, such as diabetes and cardiovascular diseases [1–3]. In China, the prevalence of overweight and obesity has dramatically increased in adults [4], while levels of physical fitness have decreased over the past years [5].

Typically, obesity has been classified by means of the body mass index (BMI), due to its simplicity and validation in multiple epidemiologic studies [6]. However, it has previously been shown that a quarter of children with excess body fat percentage were misdiagnosed solely based on BMI [7]. This might be especially due to the BMI not allowing to distinguish between fat and lean tissue, particularly in individuals with a BMI ≤ 30 kg/m^2^ but excessive body fat [8–11]. Interestingly, individuals with a normal BMI but a high percentage of body fat (> 20% in men and > 30% in women, respectively) bear an increased risk for systemic inflammation, metabolic dysregulation, and mortality [12]. This condition is commonly referred to as normal weight obesity (NWO) [12, 13].

However, the potential influence of NWO on health-related physical fitness is not well studied. In fact, physical fitness is considered one of the most important health markers and commonly understood as a predictor of morbidity and mortality for cardiovascular diseases [14, 15]. As such, numerous studies have shown that a high BMI and/or obesity are associated with poor physical fitness due to the higher body fat and lower skeletal muscle mass [16–19]. Moreover, both skeletal muscle mass and strength might be closely associated with cardiorespiratory fitness [20, 21] and health [22], providing reasoning to assume that skeletal muscle may mediate the relationship between NWO and physical fitness.

Consequently, gaining a better understanding of the risk factors that are associated with normal weight obesity is an important step towards designing interventions and making policies to promote healthy lifestyles in young adults. The primary aim of this study was to examine the associations of physical fitness with NWO in Chinese university students. As a secondary objective, we aimed to assess whether possible differences in physical fitness between students classified as NWO and normal weight non-obese (NWNO) are mediated by skeletal muscles mass. We hypothesized that NWO is associated with poorer physical fitness both in male and female students. In addition, it was postulated that the differences in physical fitness between NWO and NWNO groups are mediated by skeletal muscles mass.

## Methods

### Subjects

A total of 383 students (205 males and 178 females) aged 18–24 years were recruited from two universities in China during September 2015 to July 2017 to participate in the study. The data collection was carried out during the same period. All participants provided written informed consents prior to the tests and the study was approved by the Institutional Review Board of Shanghai Jiao Tong University (M15018).

### Anthropometrics and body composition

Body height was measured to the nearest 0.1 cm using a wall-mounted height scale. Body weight, total fat, visceral fat area and skeletal muscle mass were measured after emptying the bladder and in light underwear using a calibrated InBody 720 bio-impedance device (Biospace, Co, Ltd., Seoul, Korea) [23, 24]. All participants received similar instructions prior to the assessment of body composition and were required to be in a fasted state. Waist circumference was measured twice to the nearest 0.1 cm (or three times if a discrepancy of more than 3 cm was observed) at the midpoint between the lower costal margin and the level of the anterior superior iliac crests, by a flexible measuring tape.

BMI is calculated as the body mass (kg) divided by the square of the body height (m). Based on the BMI value, the participants were categorized into the three following groups: underweight (BMI < 18.5 kg/m^2^), normal weight (18.5 ≤ BMI ≤ 23.9 kg/m^2^) and overweight (BMI ≥ 24 kg/m^2^) [25]. According to the cut-off points of body fat percentage (i.e. 20% for men and 30% for women) [13], the normal weight group was further divided into a normal weight obesity and normal weight non-obese (NWNO) group.

### Self-reported physical activity

Daily physical activity was assessed by the Chinese version of the Physical Activity Questionnaire [26], which included information about the frequency, duration and intensity of each physical activity per week.

### Physical fitness

In order to allow for familiarization with all testing procedures, all participants received oral instructions and demonstrations for all physical fitness tests prior to the evaluation.

#### Cardiovascular fitness

Initially, resting systolic and diastolic blood pressure (SBP & DBP) were measured in a seated position by an Electronic sphygmomanometer (OMRON HEM 7051, Dalian, Liaoning, China). The measurement was performed twice at the interval of 2 min by a trained researcher. The mean of the two measures was used for statistical analysis.

Thereafter, a 10-min intermittent shuttle run test (Andersen test) [27] was performed to estimate cardiovascular fitness. During this test, the participants alternate between running and standing for 15 s at a time. The participants were required to cover as much distance as possible for 5 min [27]. The total running distance covered was used for statistical analysis.

#### Muscular power

Muscular power of the lower extremities was assessed by a countermovement jump (CMJ) with arm swing [28, 29]. The test was repeated 3 times at interval for 2 min. Jumping height was assessed by a flexible measuring tape attached to the level of the 4-5th lumbar vertebra. After correctly preparing the equipment, the participants were firstly instructed to squat and jump as quickly and high as possible using the arms. The difference between the initial and final values of the ruler at the junction of the belt and ground tape was used to determine jumping height. The average jump height of all 3 trials was used for statistical analysis.

#### Speed and agility

Speed and agility were assessed by a 5 × 5 m shuttle run test (5mSR) [30, 31]. During this test, the participants were requested to alternate with maximal movement velocity between two lines, each 5 m apart. Participants were required to cross both lines entirely with their feet, while back and forth lap was counted as one lap. This was repeated 5 times without rest. The total time of the best completed set of laps was used for statistical analysis.

Based on the results of all three tests, we also generated a gender-specific composite physical fitness score, defined as the sum of the standardized values (z-score) of all three tests [32, 33].

### Statistical analysis

All statistical analyses were performed separated by gender. Normality of the data was checked by calculating skewness and kurtosis and also tested by the Kolmogorov–Smirnov test. Baseline characteristics were presented using means and standard deviations. Weight-group differences in baseline characteristics were assessed using independent T-tests. In addition, analysis of covariance (ANCOVA) was used to examine the between-group differences on physical fitness with adjustment for age and physical activity. These analyses were performed using SPSS 22.0 (SPSS Inc., Chicago, IL, USA) with a two-sided significance level of 0.05.

**Fig. 1:**
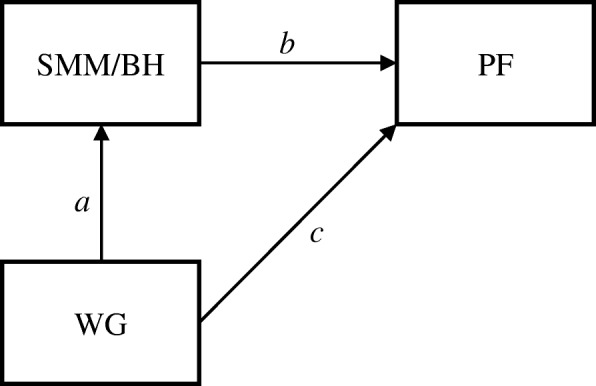
Conceptual model of the mediation analysis. (*a* representing the effect of weight group (WG) on body height-adjusted skeletal muscle mass (SMM/BH), *b* the effect of SMM/BH on physical fitness (PF), and *c* the effect of weight group on physical fitness (PF))

Mediation analyses were conducted in order to assess whether skeletal muscle mass mediated the relationship between weight group and physical fitness (Andersen test, CMJ, 5mSR, sum of physical fitness), adjusted for age and physical activity. The mediation analysis was conducted by Mplus version 7 (Muthén & Muthén, Los Angeles, CA, USA). As shown in Fig. 1, in the mediation models weight group (NWO vs. NWNO) was considered as an independent variable, while body height-adjusted skeletal muscle mass (SMM/BH) used as the mediator variable and measures of physical fitness (Andersen test, CMJ, 5mSR, sum of physical fitness) were treated as dependent variables. According to Imai [34] and Loeys [35], the following criteria must be met to establish mediation effects. First, the independent variable must significantly influence the mediator; second, the independent variable must significantly influence the dependent variable; third, the mediator must significantly influence the dependent variable. Further, if the effect of the independent variable on the dependent variable (i.e. direct effect) entirely diminishes when the mediator is included in the model, the mediator fully mediates between independent and dependent variables (full mediation). If the effect of the independent variable on the dependent variable remains with a smaller magnitude when the mediator is included in the model, the mediator partially mediates between independent and dependent variables (partial mediation) [36]. Direct, indirect and total effects from the models as well as the proportion of variance of the physical performance variables explained by the model were reported. The proportion of effect mediation was calculated by the equation = 100 * indirect effect / total effect [36].

## Results

### Participants’ characteristics

Out of the 383 participants, 14.1% of males and 36.0% of females were classified as underweight, and 61.0% and 59.5% as normal weight, respectively. In addition, 24.9% of males and 4.5% of females were classified as overweight. Of the participants who were classified as normal weight, 47.3% of males and 32.0% of females were classified as NWNO, while 13.7% of males and 27.5% of females were NWO. The characteristics of all the participants are shown in Additional file 1: Table S1. For the purpose of the present study, the analyses were restricted to the NWO and NWNO groups.

The participants’ characteristics of NWO and NWNO are shown in Table 1. Most of the anthropometric and physical characteristics differed significantly between genders (all *p <* 0.05), except for age (*p =* 0.20) and diastolic blood pressure (*p =* 0.47). No significant difference was observed between NWO and NWNO in body weight and heart rate in both genders and physical activity in male students (all *p* > 0.05). Age, waist circumference, visceral fat area, body fat percentage and BMI were significantly higher in NWO compared to NWNO both in male and female students (all *p* < 0.05) and physical activity only in females (*p* = 0.03). Body height and skeletal muscle mass were significantly lower in NWO than NWNO for both genders (all *p* < 0.05). Furthermore, DBP and SBP were significantly higher in NWO compared to NWNO only in females (all *p* < 0.001).

**Table 1:** Participants characteristics stratified by gender and weight group (Mean ± SD)

Variables	Men (*n* = 125)		Women (*n* = 106)	
	NWNO (*n* = 97)	NWO (*n* = 28)	NWNO (*n* = 57)	NWO (*n* = 49)
Age (year)	20.2 ± 1.4*	20.9 ± 1.8	19.7 ± 1.6**	20.6 ± 1.5
BH (cm)	175.8 ± 6.5**	171.5 ± 6.8	162.9 ± 6.2*	160.0 ± 5.1
BW (Kg)	65.3 ± 6.6	66.1 ± 6.2	53.9 ± 5.5	55.5 ± 4.6
WC (cm)	75.0 ± 4.4**	79.7 ± 4.0	68.1 ± 4.4**	73.2 ± 4.5
VFA (cm^2^)	28.5 ± 13.7**	59.7 ± 13.3	52.4 ± 9.1**	73.2 ± 14.3
Fat %	12.3 ± 3.8**	23.1 ± 3.4	24.4 ± 2.6**	32.1 ± 2.3
BMI (kg/m^2^)	21.1 ± 1.4**	22.5 ± 1.2	20.3 ± 1.3**	21.7 ± 1.3
SMM/BH (Kg/m)	18.3 ± 1.5**	16.5 ± 1.5	13.5 ± 1.1**	12.6 ± 0.9
DBP (mmHg)	69.5 ± 7.0	71.9 ± 8.8	66.9 ± 6.1**	72.1 ± 8.4
SBP (mmHg)	120.4 ± 9.5	119.6 ± 10.0	104.9 ± 8.3**	112.3 ± 10.7
HR	72.7 ± 9.8	73.7 ± 12.5	75.5 ± 9.4	75.8 ± 9.7
PA (Mets*H/wk)	30.4 ± 10.4	27.8 ± 13.1	21.7 ± 8.6*	27.5 ± 15.3

*Abbreviations*: *NWNO* Normal weight non-obese, *NWO* Normal weight obesity

*BH* Body height, *BW* Body weight, *WC* Waist circumference, *VFA* Visceral fat area, *Fat%* Body fat percentage, *BMI* Body mass index, *SMM* Skeletal muscle mass, *DBP* Diastolic blood pressure, *SBP* Systolic blood pressure, *HR* Heart rate, *PA* Physical activity;

*Indicates *p* < 0.05 (NWNO vs. NWO)

**Indicates *p* < 0.01 (NWNO vs. NWO)

### Differences in physical fitness between NWO and NWNO

**Fig. 2:**
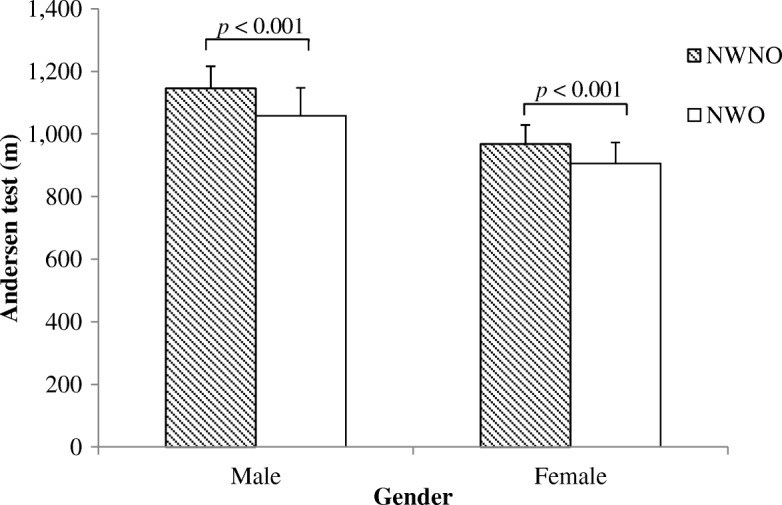
Distance covered during the Andersen Test in NWO and NWNO

**Fig. 3:**
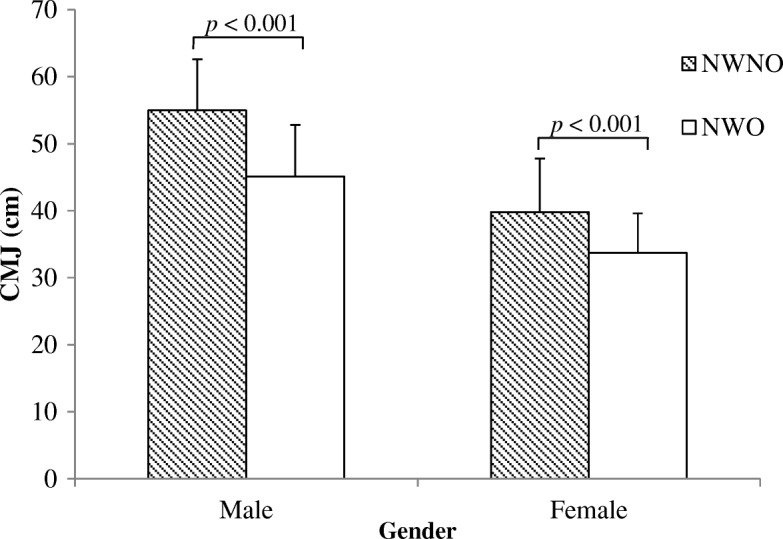
Countermovement jumping (CMJ) height in NWO and NWNO

**Fig. 4:**
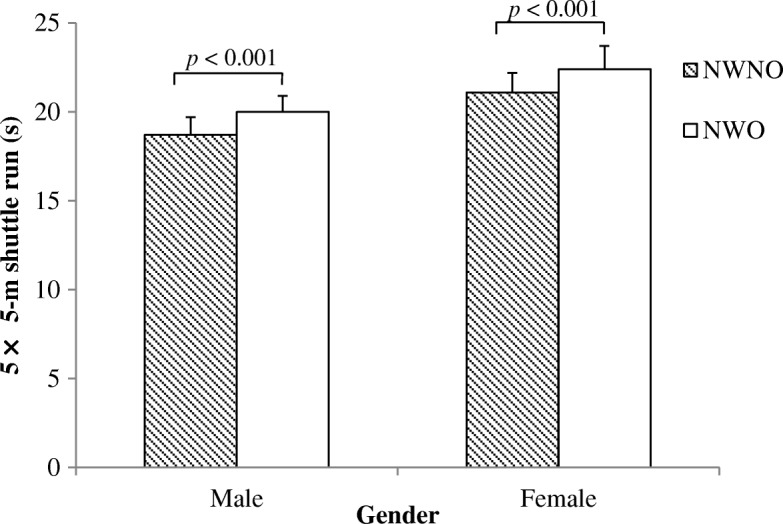
Time needed to cover the 5 × 5-m shuttle run test in NWO and NWNO

**Fig. 5:**
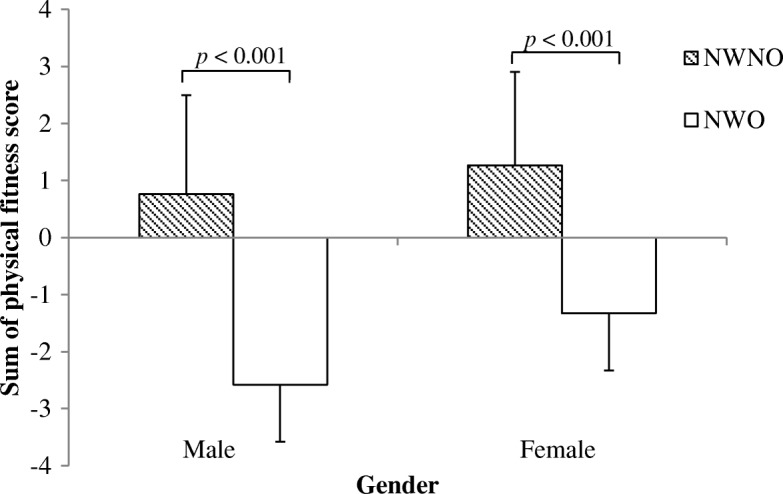
Sum of physical fitness score in NWO and NWNO

After adjustment for age and physical activity, significant differences were observed between groups in all three measures of physical fitness. The three physical fitness measures were similar between NWO and overweight (OW) groups (Additional file 1: Table S1). However, compared to NWNO, participants classified as NWO showed a poorer performance in the Andersen test (males: 1146 ± 70 m vs. 1046 ± 95 m, females: 968 ± 61 m vs. 907 ± 67 m, *p* < 0.001), CMJ (males: 55 ± 7.6 cm vs. 44.9 ± 7.5 cm, females: 39.8 ± 8.0 cm vs. 33.7 ± 5.9 cm, *p* < 0.001) and 5mSR (males: 18.7 ± 1.0 s vs. 20.0 ± 0.9 s, females: 21.1 ± 1.1 s vs. 22.4 ± 1.3 s, *p* < 0.001), as well as in the sum of the physical fitness score (males: 0.8 ± 1.7 vs. -2.6 ± 1.9, females: 1.3 ± 1.6 vs.-1.3 ± 1.7, *p* < 0.001), respectively (Figs. 2, 3, 4 and 5).

### Results of the mediation analyses

Our study fulfilled the three criteria for significant mediation effects between 1) weight group and each outcome, 2) weight group and skeletal muscle mass, and 3) weight group and skeletal muscle mass predicting each outcome, as well as the effect modification of weight group from model 1) to 3) in the appropriate direction (i.e. the weight group had a lower effect in model 3) for all cases except CMJ among girls. The path coefficients of direct, indirect and total effects of the mediation models are shown in Table 2. For cardiovascular fitness, there was an indirect effect of weight group on performance in the Andersen test through skeletal muscle mass both in male (*p* < 0.01) and female students (*p* < 0.05, proportion of mediation: men 27%, women 23%). The direct effect of weight group was also significant (*p* < 0.001), indicating that skeletal muscle mass partially mediated the negative relationship between the body weight group and cardiovascular fitness in both male and female students. Similarly, also in speed and agility an indirect effect of the weight group on performance in the 5mSR test through skeletal muscle mass was observed both in male and female students (*p* < 0.01, proportion of mediation: men 24%, women 23%). The direct effect of weight group on 5mSR performance was also significant (*p* < 0.001), which indicated that skeletal muscle mass partially mediated the negative relationship between body weight group and speed and agility in male and female students.

**Table 2:** Standardized effects of the mediation models by gender

Model (male)	Path coefficients	95% CI	*p*-value	Model (female)	Path coefficients	95% CI	*p*-value
Andersen test (A)				Andersen test (A)			
Direct effects				Direct effects			
WG → SMM (a)	−0.46	− 0.60~ − 0.32	< 0.001	WG → SMM (*a*)	−0.38	− 0.54~ − 0.22	< 0.001
SMM → A (*b*)	0.28	0.10~0.45	0.002	SMM → A (*b*)	0.25	0.06~0.45	0.012
WG → A (c)	−0.36	−0.53~ − 0.19	< 0.001	WG → A (c)	−0.33	− 0.53~ − 0.14	0.001
Indirect effects				Indirect effects			
WG → A *(a* × b)	− 0.13	−0.22~ − 0.04	0.006	WG → A *(a* × b)	−0.10	− 0.18~ − 0.01	0.029
Total effect				Total effect			
WG → A *(a* × *b* + *c*)	−0.49	− 0.63~ − 0.34	< 0.001	WG → A *(a* × *b* + c)	−0.43	− 0.61~ − 0.25	< 0.001
*R*^*2*^(A)	0.30	0.14~0.46	< 0.001	*R*^*2*^(A)	0.24	0.08~0.40	0.003
CMJ (C)				CMJ (C)			
Direct effects				Direct effects			
WG → SMM (*a*)	−0.46	−0.60~ − 0.32	< 0.001	WG → SMM (*a*)	− 0.38	−0.54~ − 0.22	< 0.001
SMM → C (*b*)	0.30	0.13~0.47	< 0.001	SMM → C (*b*)	0.17	−0.02~0.35	0.074
WG → C (c)	−0.36	− 0.52~0.20	< 0.001	WG → C (c)	− 0.34	−0.52~ − 0.16	< 0.001
Indirect effects				Indirect effects			
WG → C *(a* × b)	− 0.14	− 0.23~ − 0.05	0.002	WG → C *(a* × b)	− 0.06	− 0.14~0.01	0.098
Total effect				Total effect			
WG → C *(a* × *b* + *c*)	− 0.50	− 0.63~ − 0.36	< 0.001	WG → C *(a* × *b* + c)	− 0.40	−0.56~ − 0.24	< 0.001
*R*^2^(C)	0.32	0.18~0.46	< 0.001	*R*^2^(C)	0.18	0.04~0.32	0.007
5mSR (S)				5mSR (S)			
Direct effects				Direct effects			
WG → SMM (*a*)	−0.46	−0.60~ − 0.32	< 0.001	WG → SMM (*a*)	− 0.38	−0.54~ − 0.22	< 0.001
SMM → S (*b*)	− 0.27	−0.43~ − 0.10	0.002	SMM → S (*b*)	−0.30	− 0.48~ − 0.12	0.001
WG → S (c)	0.37	0.21~0.53	< 0.001	WG → S (c)	0.36	0.18~0.53	< 0.001
Indirect effects				Indirect effects			
WG → S *(a* × b)	0.12	0.04~0.21	0.006	WG → S *(a* × b)	0.11	0.03~0.20	0.007
Total effect				Total effect			
WG → S *(a* × *b* + *c*)	0.49	0.35~0.62	< 0.001	WG → S *(a* × *b* + c)	0.47	0.32~0.63	< 0.001
*R*^*2*^(S)	0.29	0.15~0.43	< 0.001	*R*^2^(S)	0.30	0.14~0.46	< 0.001
SumPF (S_PF_)				SumPF (S_PF_)			
Direct effects				Direct effects			
WG → SMM *(a)*	−0.46	−0.60~ − 0.32	< 0.001	WG → SMM (a)	− 0.38	− 0.54~ − 0.22	< 0.001
SMM → S_PF_*(b)*	0.32	0.16~0.48	< 0.001	SMM → S_PF_ (*b*)	0.34	0.17~0.51	< 0.001
WG → S_PF_ (c)	− 0.49	− 0.63~ − 0.34	< 0.001	WG → S_PF_ (c)	−0.48	− 0.64~ − 0.32	< 0.001
Indirect effects				Indirect effects			
WG → S_PF_*(a* × b)	− 0.15	−0.23~ − 0.06	0.001	WG → S_PF_*(a* × b)	−0.13	− 0.21~ − 0.05	0.003
Total effect				Total effect			
WG → S_PF_*(a* × *b* + *c*)	−0.63	− 0.74~ − 0.52	< 0.001	WG → S_PF_*(a* × *b* + c)	− 0.61	−0.75~ − 0.47	< 0.001
*R*^2^(S_PF_)	0.48	0.34~0.62	< 0.001	*R*^2^(S_PF_)	0.47	0.31~0.63	< 0.001

*Abbreviations*: *WG* Weight group, *SMM* Skeletal muscle mass, *CMJ* Countermovement jump, *5mSR* 5 × 5-m shuttle run, *SumPF* Sum of physical fitness score, *95% CI* 95% confidence intervals

There was an indirect effect of weight group on performance in CMJ test through skeletal muscle mass only in male students (*p* = 0.002, proportion of mediation: 28%). The direct effect of weight group was also significant (*p* < 0.001), indicating that skeletal muscle mass partially mediated the negative relationship between the body weight group and muscular power in male students.

For the sum score of physical fitness, the indirect and direct effects were both statistically significant in male and female students (all *p* < 0.01, proportion mediation: male 24%, female 21%), which indicates that the negative relationship between body weight group and physical fitness was partially mediated by skeletal muscle mass. Thus, the NWO university students tended to have less skeletal muscle mass, which might partly contribute to the observed decreased physical fitness.

## Discussion

The primary findings of the study were that 13.7% of the male and 27.5% of the female students were classified as NWO and these students were characterized by lower levels of physical fitness compared with their NWNO peers. Furthermore, we found that low levels of physical fitness (SumPF, Andersen test, 5mSR) in NWO were partially mediated by a lower skeletal muscle mass in both males and females. For CMJ, however, the mediation role of skeletal muscle mass was observed only in male students.

The high prevalence of NWO in both male and female students was in contrast to a Swiss study in adults (aged 35–75 years), which showed a much lower prevalence of NWO of only 5.4% in women and less than 3% in men [37]. However, in a Finnish study of young women (mean age = 18 years), the prevalence of NWO was estimated to be 39.0% [38, 39]. Our results concerning the prevalence of NWO in women were similar to a study of normal weight central obesity in South Africa [40]. The differences among these studies are somewhat interesting but it should be noted that the results cannot be directly compared, since to the best of our knowledge, our study is the first to investigate the prevalence of NWO among Chinese university students. The large discrepancy between studies may be attributed to the differences in the assessment methods used and participant characteristics such as age, dietary characteristics, lifestyle, and ethnicity. Despite these differences between the present study and previous investigations, our results indicated a potential hidden health threat by which nearly every eighth male and especially every third female student in China could be classified as normal weight obese.

Previous studies have shown that NWO is associated with metabolic disorders [37, 41], an increased risk of the metabolic syndrome [42] and cardiovascular diseases, as well as an increase in all-cause mortality [43]. However, little is known about the association of NWO with physical fitness. The findings of the current study extend to previous work by showing that young adults with a normal BMI but excessive body fat mass performed worse on the three measures of physical fitness. Since lower physical fitness is a predictor of chronic diseases [44], the findings of the current study are clinically important. Thus, students classified as NWO should not only follow standard physical activity recommendations but should also target on progressive muscular strength training to improve physical fitness and prevent further potential health risks. Importantly, although the current study focused on the NWO in young adults, this may not necessarily mean that the established health risks of overweight and obesity can be neglected. However, given the potential influence of NWO on physical fitness, the findings of our study highlight the importance of considering body composition analysis rather than only measuring BMI in future preventive studies and practices.

In order to clarify the roles of skeletal muscle mass on the relationship between NWO and physical fitness, we further conducted a series of mediation analyses and found that NWO had a negative influence on skeletal muscle mass and measures of physical fitness. In accordance with our hypotheses, the between–group difference on the sum score of physical fitness, cardiovascular fitness and the 5mSR test between NWO and NWNO were partially mediated by skeletal muscle mass in both male and female university students. Previous studies have shown that excessive adiposity is associated with lower cardiorespiratory fitness in adults, while skeletal muscle mass is correlated with cardiorespiratory fitness [21, 45]. Therefore, lower skeletal muscle mass may at least partially account for the poorer physical fitness in students with NWO. The physiological reasoning of our findings may be manifold but have previously been linked to a delayed onset of fatigue due to increased muscle mass and strength [46] as well as an improved central circulation due to an improved venous return [47]. Interestingly, in jumping height a significant partial mediation effect of skeletal muscle mass was observed only in male students. However, while not statistically significant, the direction of the indirect effect of skeletal muscle mass on CMJ was similar in female students; in fact it was less than 0.1 and pointed to the same direction as in men. Therefore, the present analysis should be reproduced in a larger sample to validate whether in fact the paths are actually similar in both genders. Taken together, skeletal muscle mass is a mediator for the physical fitness in students with normal weight but high fat mass.

When interpreting the present findings, some strengths and limitations need to be considered. The classifications of NWO were based on Chinese-specific cut-off points for BMI, which increases the generalizability and comparability of the findings to the Chinese population but not necessarily to other nations. In addition to analyzing the relationship between NWO and physical fitness, we further performed a mediation analysis to clarify whether or not the observed relationships are explained by the differences in skeletal muscle mass. However, one should bear in mind that the cross-sectional design of the study does only allow to draw associations but not causalities [48]. Thus, longitudinal studies are needed to confirm the observed direction of the relationship between body composition and physical fitness. In addition, while using muscle mass as a mediator is well justified based on previous studies [20–22], we recommend to utilize precise measures of physical activity (e.g. objectively assessed physical activity) in future studies in order to clarify the role of physical activity in NWO. Last, the study population included only university students. While this is an important population to study, further investigations are needed to confirm the findings in broader age groups. Despite these limitations, the results are of relevance because so far only few studies have yet examined the effects of physical fitness in relation to normal weight obesity.

## Conclusion

This study showed that 13.7% of male and 27.5% of female students were classified as NWO and these students were characterized by lower levels of physical fitness than their NWNO peers. The low levels of physical fitness in NWO were partially explained by a lower skeletal muscle mass in both males and females. The findings indicated that much attention should be paid for the potential hidden health risk in university students with normal body mass index but excessive fat mass.

## Additional file

Additional file 1: Participants characteristics stratified by gender and four weight group. (PDF 129 kb)

## Abbreviations

BMI: Body mass index
NWO: Normal weight obesity
NWNO: Normal weight non-obese
BH: Body height
BW: Body weight
WC: Waist circumference
VFA: Visceral fat area
Fat%: Body fat percentage
SMM: Skeletal muscle mass
DBP: Diastolic blood pressure
SBP: Systolic blood pressure
HR: Heart rate
PA: Physical activity
WG: Weight group
SMM: Skeletal muscle mass
CMJ: Countermovement jump
5mSR: 5 × 5 m shuttle run
SumPF: Sum of physical fitness score
95% CI: 95% confidence intervals
