# Supplementary material for: Normal weight obesity and physical fitness in Chinese university students: an overlooked association
Source: BMC Public Health. 2018 Dec 4;18:1334. doi: 10.1186/s12889-018-6238-3 (PMC6278052; doi:10.1186/s12889-018-6238-3)
Supplement: Supplementary file 1 — Participants characteristics stratified by gender and four weight group. (PDF 129 kb) [file 12889_2018_6238_MOESM1_ESM.pdf]

Additional file 1: Participants characteristics stratified by gender and four weight group (Mean ± SD).

Variables	Men (n=205)				Women (n=178)			
	UW(n=29)	NWNO(n=97)	NWO(n=28)	OW(n=51)	UW(n=64)	NWNO(n=57)	NWO(n=49)	OW(n=8)
Age (year)	20.1 ±1.4	20.2 ±1.4	20.9 ±1.8	21 ±1.6	20.1 ±1.7	19.7 ±1.6	20.6 ±1.5	20 ±1.5
BH (cm)	174.6 ±6.1	175.8 ±6.5	171.5 ±6.8	172.8 ±6	162.1 ±5.5	162.9 ±6.1	160 ±5.1	162.7 ±5.6
BW (Kg)	53.2 ±4.5	65.3 ±6.6	66.1 ±6.2	78.2 ±7.6	46.3 ±3.7	53.9 ±5.5	55.5 ±4.6	65.5 ±4.6
WC (cm）	67 ±3.1	75 ±4.4	79.7 ±4	87.3 ±5.9	63.6 ±3.5	68.1 ±4.4	73.2 ±4.5	81.3 ±5.1
VFA (cm^2)	12.1 ±6.5	28.5 ±13.7	59.7 ±13.3	68.8 ±12.2	38.9 ±9.6	52.4 ±9.1	73.2 ±14.3	78.9 ±0.2
Fat %	9.6 ±2.8	12.3 ±3.8	23.1 ±3.4	23.6 ±5.2	21.7 ±4.5	24.4 ±2.6	32.1 ±2.3	36.2 ±3
BMI (kg/m^2)	17.4 ±0.9	21.1 ±1.4	22.5 ±1.2	26.2 ±1.7	17.6 ±0.7	20.3 ±1.3	21.7 ±1.3	25 ±0.5
SMM/BH (Kg/m)	15.3 ±1.1	18.3 ±1.5	16.5 ±1.5	19.5 ±2	11.9 ±1	13.5 ±1.1	12.6 ±0.9	13.9 ±0.9
DBP (mmHg）	70.7 ±6.8	69.5 ±7.0	71.9 ±8.8	72.1 ±8.0	68.1 ±7.8	66.9 ±6.1	72.1 ±8.4	71.1 ±7.2
SBP (mmHg）	117.2 ±10.1	120.4 ±9.5	119.6 ±10.0	126.1 ±9.4	105.9 ±8.3	104.9 ±8..3	112.3 ±10.7	110.2 ±5.7
HR	78.3 ±12.7	72.7 ±9.8	73.7 ±12.5	73.1 ±10.9	77.7 ±11.4	75.5 ±9.4	75.8 ±9.7	73.8 ±8.3
PA (Mets*H/wk)	27.9 ±16.3	30.4 ±10.4	28 ±13	35.1 ±14.3	23.5 ±14.5	21.7 ±8.6	27.5 ±15.3	30.1 ±10.4
Anderson test (m)	1103 ±90.4	1145.8 ±70.2	1045.9 ±95.4	1049.2 ±78.3	923.3 ±97.4	967.8 ±61.3	906.6 ±66.5	866.5 ±49.3
CMJ (cm)	53.5 ±7.5	55 ±7.6	44.9 ±7.5	48.6 ±7.7	34.9 ±8	39.8 ±8	33.7 ±5.9	31.7 ±5.2
5mSR (s)	19.3 ±1.4	18.7 ±1	20 ±0.9	19.6 ±1	21.7 ±1.4	21.1 ±1.1	22.4 ±1.3	22.5 ±1.3

Abbreviations: UW=Under Weight; NWNO=Normal Weight Non-obese; NWO=Normal Weight Obese; OW=Over-weight.

BH=Body height; BW=Body weight; WC=Waist circumference; VFA=Visceral fat area; Fat%=Body fat percentage; BMI=Body Mass Index; SMM=Skeletal muscle mass; DBP=Diastolic blood pressure; SBP=Systolic blood pressure; HR=Heart rate; PA=Physical activity; CMJ=Countermovement jump; 5mSR=5 × 5-m shuttle run test.
